# Additional fiber orientations in the sagittal stratum—noise or anatomical fine structure?

**DOI:** 10.1007/s00429-021-02439-w

**Published:** 2022-02-03

**Authors:** Svenja Caspers, Markus Axer, David Gräßel, Katrin Amunts

**Affiliations:** 1 Institute for Anatomy I, Medical Faculty & University Hospital Düsseldorf, Heinrich-Heine-University Düsseldorf, 40225 Düsseldorf, Germany; 2 Institute of Neuroscience and Medicine (INM-1), Research Centre Jülich, 52425 Jülich, Germany; 3 C. and O. Vogt Institute for Brain Research, Medical Faculty & University Hospital Düsseldorf, Heinrich Heine University Düsseldorf, 40225 Düsseldorf, Germany

**Keywords:** Visual system, Optic radiation, Callosal fibers, Diffusion imaging, Spherical deconvolution, 3D-PLI

## Abstract

The sagittal stratum is a prominent and macroscopically clearly visible white-matter structure within occipital and parietal lobes with a highly organized structure of parallel fibers running in rostro-caudal direction. Apart from the major tract running through, i.e., the optic radiation, the source and arrangement of other fibers within the sagittal stratum is only partially understood. Recent diffusion imaging studies in-vivo suggest additional minor fiber directions, perpendicular to the major rostro-caudal ones, but the spatial resolution does not allow to resolve them, and to unambiguously distinguish it from noise. Taking this previous evidence as motivation, the present study used 3D polarized light imaging (3D-PLI) for micrometer resolution analysis of nerve fibers in postmortem specimens of a vervet monkey brain. The analysis of coronal occipital and parietal sections revealed that the sagittal stratum consisted of an external and an internal layer, which are joined and crossed by fibers from the surrounding white matter and the tapetum. Fibers from different parietal and occipital regions entered the sagittal stratum in the dorsal, ventral or middle sector, as solid large bundles or as several small fiber aggregations. These patterns were remarkably similar to published results of tracer experiments in macaques. Taking this correspondence as external validation of 3D-PLI enabled translation to the human brain, where a similarly complex fiber architecture within the sagittal stratum could be exemplified in a human hemisphere in our study. We thus argue in favor of a dedicated fiber microstructure within the sagittal stratum as a correlate of the additional fiber directions typically seen in in-vivo diffusion imaging studies.

## Introduction

**Fig. 1:**
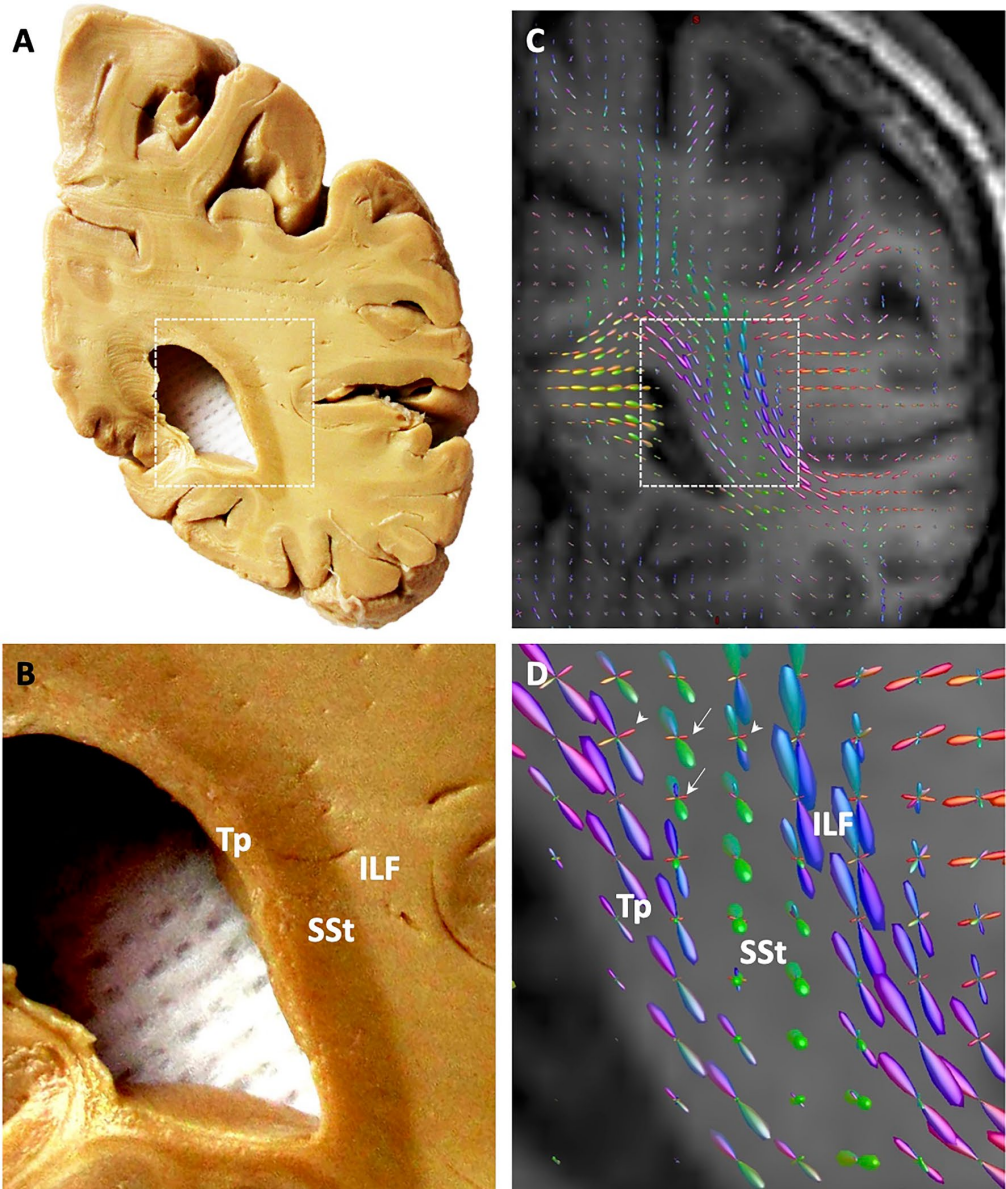
Sagittal stratum and its fibers in the human brain. **A** Coronal slab through the transition between parietal and occipital lobes, from dissection of a post-mortem human brain. **B** Higher magnification of the slab. **C** Orientation distribution functions (ODFs) exemplarily representing the main fiber directions within each voxel of a comparable brain section as in **A**. ODFs were calculated from in-vivo high-angular resolution diffusion imaging data (obtained on a 3 T Tim-TRIO Siemens MR scanner; 120 directions, *b* = 2700 s/mm^2^, voxel resolution: 2.4 × 2.4 × 2.4 mm^3^) of a subject from the population-based 1000BRAINS study (Caspers et al. 2014), using the constrained spherical deconvolution approach (Tournier et al. 2007) as implemented in the MRtrix software package (Tournier et al. 2012). Note the additional secondary fiber directions within the ODFs of the sagittal stratum (SSt), indicated by white arrows, and at the transition from the SSt into the tapetum (Tp) and the inferior longitudinal fascicle (ILF), indicated by white arrow heads in (**D**)

The term sagittal stratum (SSt) is describing a composite of fiber bundles within the deep white matter of the occipital and adjacent temporal and parietal lobes. It is clearly visible on coronal brain sections from postmortem dissection due to the course of the highly parallel fibers, which run mainly in rostro-caudal direction (Fig. 1A, B). It appears as a dark stripe within the surrounding white matter, the latter being composed of the tapetum medially, containing the callosal fibers of the visual areas (Caspers et al. 2015), and the large association fiber bundles, such as the inferior and superior longitudinal and fronto-occipital fascicles laterally (Catani et al. 2003; Cristina et al. 2014; Forkel et al. 2015; Takemura et al. 2020).

Disentangling the fibers running within the sagittal stratum and their microarchitecture has been a matter of debate for a long time. While it was largely agreed upon that the optic radiation would be at least part of the sagittal stratum, it was debated if it contained other projection or even association fibers (Schmahmann and Pandya 2006). Sachs (1892) and Flechsig (1896) showed that the optic radiation only covered one part of the sagittal stratum, i.e., the external part (SSte), while there was an additional internal layer of the sagittal stratum (SSti) containing other fibers (Forkel et al. 2015). In addition to that, Pfeifer (1925) was able to clearly demonstrate the bilaminar structure of the sagittal stratum using a myeloarchitectonic stain of horizontal brain sections from infant brains. A similar description of the SSte was provided by Dejerine (1895), assuming that the dominant fiber bundle herein would be the inferior longitudinal fascicle. This gave rise to the idea that the sagittal stratum might also contain association fibers. Polyak (1957) focused back on the optic radiation as the relevant fiber tract covering a major part of the sagittal stratum, which can be followed all the way from the lateral geniculate body to the occipital pole. Vergani et al. (2014) partially corroborated these findings using macroanatomic Klingler dissection, without being able to consistently identify the bilaminar structure of the sagittal stratum in all sections and throughout its whole dorso-ventral extent.

The origin and terminations of the fibers in the internal layer of the sagittal stratum were less well understood. Sachs (1892) already assumed that these might be corticofugal fibers from the occipital cortex to the thalamus and the brain stem. This has lately been supported by tracer studies in macaques, showing that fibers from different areas of the occipital and adjacent temporal and parietal cortex enter the sagittal stratum and run in its internal layer to subcortical nuclei (Schmahmann and Pandya 2006). This would require fibers to cross the SSte to reach the SSti. While this could be shown by the tracer studies for the macaque brain (Schmahmann and Pandya 2006), it was only assumed, but hardly ever seen in humans using classic macrodissection or myeloarchitectonic stains. Better resolving the fibers entering or leaving this very large bundle of tracts within the optic radiation would finally allow for disentangling different fiber populations beyond the dominant optic radiation with completely different (subcortical) targets. This would add to the understanding of feedforward and top-down feedback loops within the visual system from higher order visual occipital and parietal cortices towards thalamic nuclei and superior colliculi and vice versa (Bennett et al. 2019; Blot et al. 2021; Lynch and Tian 2006). Furthermore, this knowledge is highly desirable in accurate neurosurgical planning (Di Carlo et al. 2019).

As tracer injections over long distances are not feasible in humans, modern imaging techniques are required to further understand the fine fiber architecture in the human sagittal stratum. Using in-vivo diffusion-weighted imaging (DWI), advanced acquisition protocols and algorithms for calculation of fiber directions allow reconstructing major and minor fiber orientations within each voxel. This not only enables clear distinction of the sagittal stratum from the surrounding white matter by its predominant rostro-caudal fiber orientation. It also showed additional fiber directions perpendicular to it, much weaker than the primary rostro-caudal direction (Fig. 1C, D). As DWI is an indirect measure for obtaining fiber orientations at mm resolution, additional information about the fiber microarchitecture is warranted. This would enable to evaluate if the additional fiber orientations as revealed by in-vivo DWI represent any underlying fiber microstructure or if they are false positives due to noise in the in-vivo data.

3D polarized light imaging (3D-PLI; Axer et al. 2011a,b) enables the analysis of the architecture of axons at micrometer resolution in human postmortem brains (Caspers et al. 2015; Takemura et al. 2020; Zeineh et al. 2016; Zilles et al. 2016). Taking advantage of the ultra-high resolution of 3D-PLI, the present study analyzed the fiber architecture of the sagittal stratum, focusing on fibers entering, leaving or crossing it from the surrounding white matter. Showing this in the monkey brain allows for direct comparison of the 3D-PLI results with those from tracing experiments as the gold standard for studying structural connectivity. Agreement of the results of both techniques within the same or comparable species then paves the way for 3D-PLI analyses in the human brain, where tracing using injections is ethically not feasible.

## Materials and methods

### Preparation of brain tissue

The fiber architecture of the sagittal stratum was studied in postmortem brain sections of parietal and occipital lobes of an adult vervet monkey brain and a right human hemisphere. The vervet monkey brain (age: 2.5 years) was removed from the skull and then perfusion-fixed in accordance with the UCLA Chancellor’s Animal Research Committee (ARC #2011-135) and the Wake Forest Institutional Animal Care and Use Committee (IACUC #A11-219). The human brain hemisphere was obtained via the body donor program of the Department of Anatomy of the University of Rostock, from a female body donor (79 years, 26 h postmortem interval) without any known history of neurologic or psychiatric disease.

The human brain was fixed in 4% buffered formalin. The vervet monkey brain was perfusion fixed with 4% paraformaldehyde solution, after deep anaesthesia of the vervet with ketamine–pentobarbital. The brain was then cryoprotected with 20% glycerol before freezing the tissue blocks. Serial coronal sections through the parietal and occipital lobes, 60 µm thick each, were obtained using a large-scale cryostat microtome (Polycut CM 3500, Leica, Germany). Blockface images of the tissue blocks were obtained during sectioning using a CCD camera to have an undistorted reference image for image realignment after sectioning. This was supported by rigid registration of a fiducial marker system (ARTag), on which the frozen tissue block was mounted (Fiala 2005; Wagner and Schmalstieg 2007). Sections were then mounted on glass slides and left unstained for measurements by means of the 3D-PLI setup.

### 3D PLI: data acquisition and processing

All vervet monkey and human brain sections were measured using the 3D-PLI setup for ultra-high-resolution analysis of the fiber architecture (Axer et al. 2011a,b). Taking advantage of the birefringent property of the myelin sheath, signals were obtained that allowed to compute fiber orientation maps (FOM), in which the arrangement of nerve fibers was studied within the sagittal stratum and surrounding white matter of the occipital and parietal lobes.

**Fig. 2:**
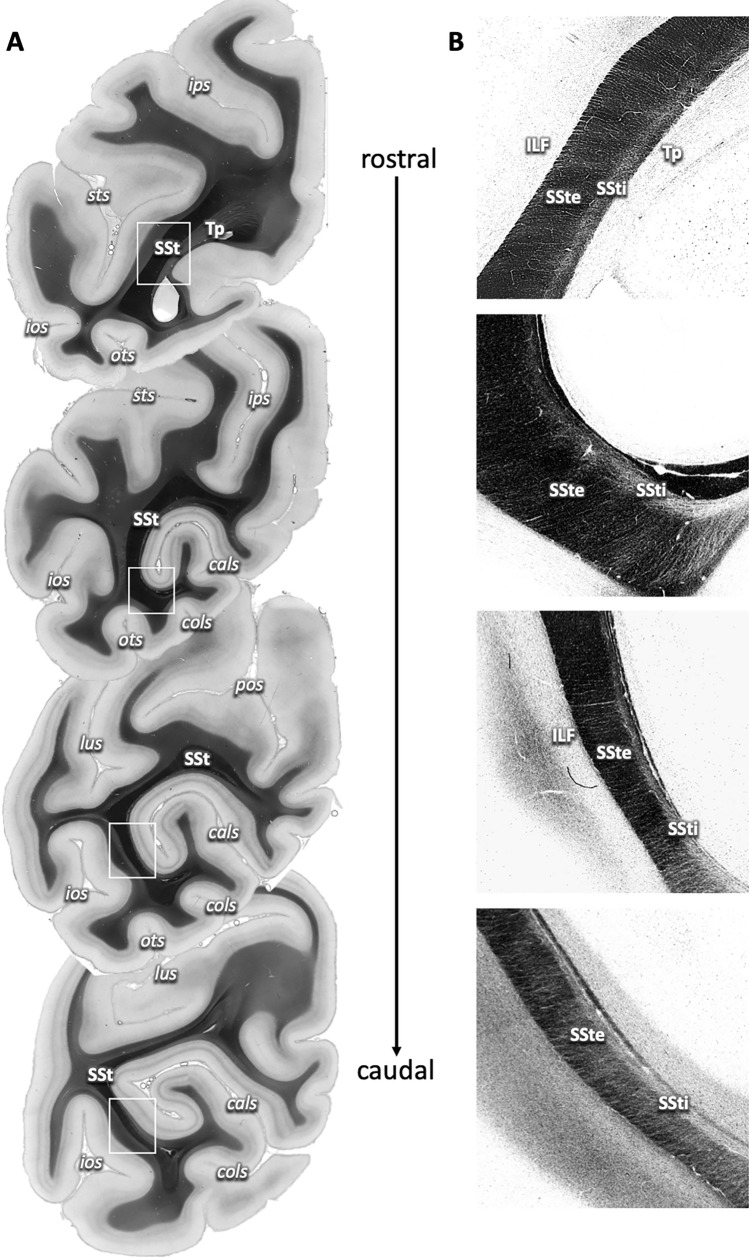
Course of the sagittal stratum (SSt) in the caudal parietal and occipital lobes on 3D-PLI transmittance maps of the vervet monkey. **A** Rostro-caudal sequence of coronal sections from caudal parietal lobe (top) to occipital pole (bottom). The SSt is visible as a thick black layer within the white matter underlying the calcarine sulcus (cals), with dorsomedial to ventrolateral orientation. White boxes indicate regions of interest as visualized in **B**. **B** Contrast-enhanced regions of interest from **A**, depicting the fine structure of the SSt as two sublayers, the external (SSte) and internal sagittal stratum (SSti) in relation to surrounding white matter, with fibers crossing and entering or leaving the sublayers. *cals* calcarine sulcus, *cols* collateral sulcus, *ios* inferior occipital sulcus, *ips* intraparietal sulcus, *lus* lunate sulcus, *ots* occipito-temporal sulcus, *pos* parieto-occipital sulcus

**Fig. 3:**
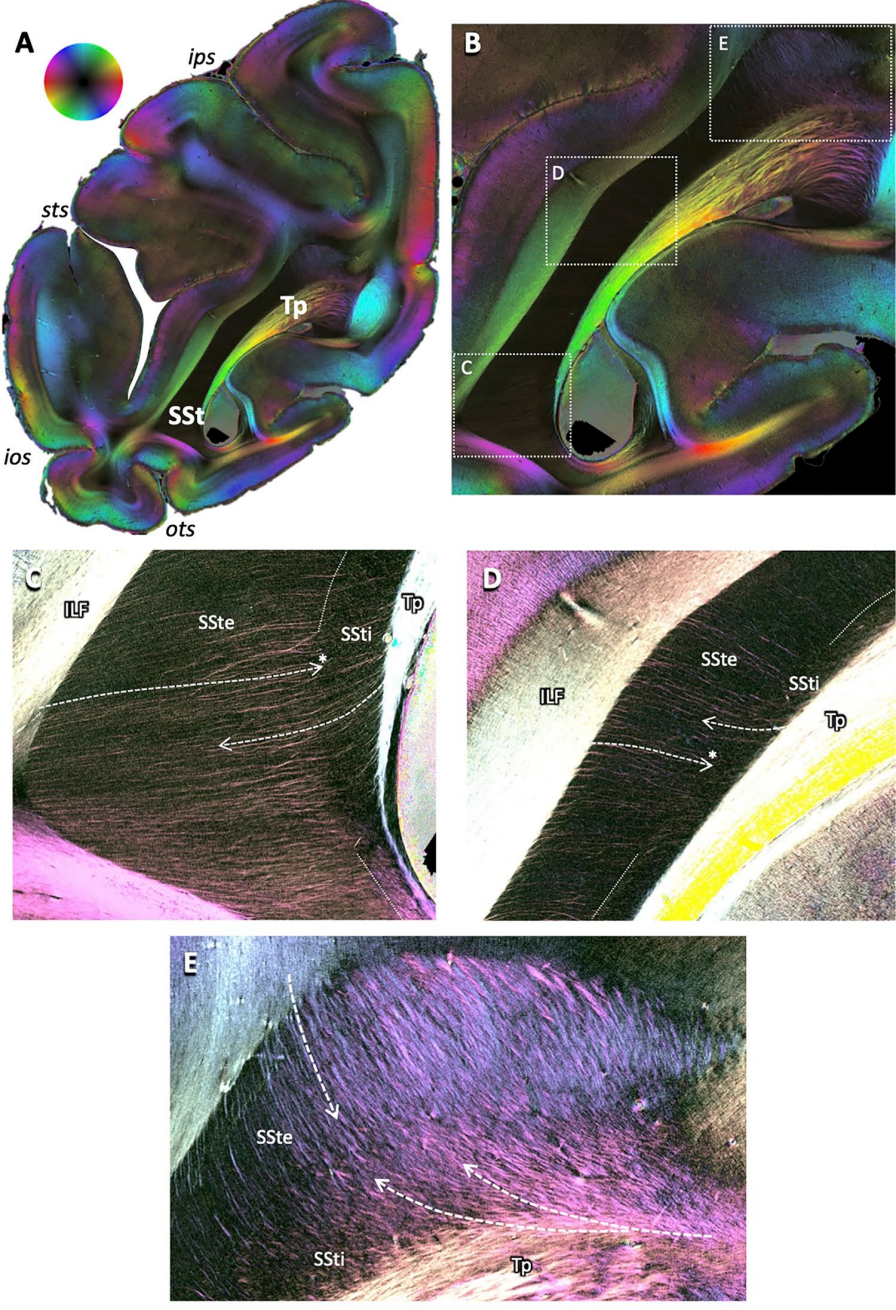
Principles of fibers crossing through or entering and leaving the sagittal stratum. **A** Fiber orientation map (FOM) of a coronal brain section of a vervet monkey brain (same as first section in Fig. 2). Fiber directions according to the color sphere at the top, with fading colors indicating change of fiber direction from within to out of the sectioning plane. **B** Enlarged view of the complete sagittal stratum. Boxes with dotted white outlines indicate regions of interest as highlighted in **C**–**E**. **C**–**E** Enlarged views of the dorsal, middle and ventral aspects of the sagittal stratum as indicated in (**B**), using enhanced color contrasts. Dashed arrows indicate courses of fibers leaving the tapetum (Tp) or leaving or entering the sagittal stratum laterally and dorsally to the surrounding white matter. Note that some fibers course through the external part of the sagittal stratum (SSte), but stop as they enter the internal part (SSti), indicating a change of direction from the sectioning plane to a course perpendicular to it (marked by asterisks at the heads of the arrows in **D** and **E**). For other conventions, see Fig. 2

Whole coronal sections of the vervet monkey brain and selected regions of interest in the human brain were scanned using a polarizing microscope (LMP-1, Taorad, Germany), with an in-plane spatial resolution of 1.3 × 1.3 µm. The polarizing microscope was equipped with a rotatable linear as well as a circular polarizer, in between which the sections were placed on a specimen stage, and a light source underneath emitting green light through this setup. Rotation of the circular polarizer allowed for image acquisition at 18 equidistant rotation angles between 0° and 170°. The final image of each section was reconstructed from a series of partially overlapping single tiles (2.7 × 2.7 mm). To extract homogeneous intensity profiles for reconstruction of the local fiber orientations, raw images were preprocessed to correct for inhomogeneities due to noise from external sources, such as illumination, dust, or filters (Dammers et al. 2010). This allowed for reconstruction of different image types by fitting the individual rotational images to a three-parameter model based on a Fourier series solution to the Jones calculus (Axer et al. 2011b), of which the transmittance, inclination and fiber orientation maps were used for the present analysis. The transmittance maps result from light scattering and extinction in the tissue, thus providing information about the overall microstructure within the section, originating from cell bodies and fibers (see Fig. 2). The transmittance maps were normalized by dynamic range. The FOMs represent the finally reconstructed and color-coded fiber orientations per pixel of a section, whereby the colors coded for fiber direction within the cutting plane, and the saturation indicating the inclination of the fibers (see color wheels, e.g., Fig. 3A). FOMs were weighted by the normalized transmittance images. As transmittance maps and FOMs are both calculated from the same preprocessed image, they are automatically coregistered, allowing for immediate comparison of different data modalities.

### Region of interest analysis

The sagittal stratum and surrounding white matter were analyzed on consecutive coronal sections, supported by an additional region-of-interest (ROI) approach to zoom into more detail. From the original whole-brain FOMs, ROIs were selected for visualization of the courses of the fibers within the sagittal stratum as well as of those entering, leaving and passing through. For visualization purposes, the ROI images of the FOMs were additionally contrast-enhanced for optimal presentation of the fiber orientation of interest. This enabled delineation of fiber tracts within consecutive sections by a trained neuroanatomist (SC), taking advantage of the very high in-plane resolution of 3D-PLI. It needs to be stressed that 3D-PLI does not provide a definite answer to the question if fibers are entering or leaving, since it does not contain information about the direction of fibers. To solve ambiguities, all results were compared to tracer studies in monkeys as provided by the present “gold standard” from Schmahmann and Pandya (2006). This allowed to interpret results obtained using 3D-PLI in the human brain, as prerequisite for mutually comparing human 3D-PLI results with diffusion-based tractography results in-vivo.

## Results

### Layering and fibers of the sagittal stratum as revealed by transmittance

The sagittal stratum was clearly identifiable on transmittance maps of consecutive coronal brain sections of the vervet monkey brain by its very dark, almost completely black appearance (Fig. 2A). From rostral to caudal, the SSt followed the occipital horn of the lateral ventricle. Its appearance changed from a straight band rostrally, running from medio-dorsal to latero-ventral and spanning the hemisphere almost from the bottom of the intraparietal sulcus (ips) to the bottom of the occipito-temporal sulcus (ots), to a C-shaped structure more caudally, which became thinner and consecutively less well delineable as it approached the occipital pole.

Zooming in revealed a clear sublayering of the SSt into a thicker external and a thinner internal layer (SSte and SSti; Fig. 2B). The SSte and SSti were identified throughout the extent of the SSt within each section as well as on all sections from rostral to caudal (except for the caudal-most sections, where the structure of the sagittal stratum dissolved). The two layers of the SSt were distinguishable by different transmittance properties: while the SSte appeared very dark (comparable to the appearance of the complete SSt in the full section views as in Fig. 2A), the SSti appeared lighter, although still considerably darker than the surrounding white matter, e.g., the inferior longitudinal fascicle (ILF) or the tapetum (Tp). In the transmittance maps, this reflected differences in fiber orientation: while the SSte was dominated by fibers coming out of the sectioning plane (thus running in rostro-caudal direction), the SSti contained more fibers running within the sectioning plane, intermingling with the rostro-caudal ones.

In addition, there were very small fiber bundles and single fibers visible in both the transmittance maps (Fig. 2B) and FOMs (Fig. 3A, B), entering or leaving the SSte or crossing through to join the SSti.

These could be subdivided into two groups: One group of fibers was entering or leaving the SSte almost perpendicularly from the laterally adjacent white matter. As these fibers approached the SSti, they changed direction to join the SSti, visible by the fading out of the fibers’ color as an indicator of steeper fiber inclination out of the sectioning plane (Fig. 3C, D; asterisks at arrow heads). The second group of fibers came out of the tapetum and revealed a straight course from the medially located tapetum through the SSti and SSte. These fibers intermingled with those from the first group, but to different degrees: while there was pronounced mixture of fiber directions in the dorsal-most part of the SSt in both SSte and SSti (Fig. 3E), the fibers of the two groups seemed to be arranged in parallel to each other, a fiber of one group running in between two fibers of the other group (Fig. 3C, D).

### Origin of fibers joining the sagittal stratum

The origin of fibers entering the SSt is known from tracer studies in monkeys. We used this information for exemplary tracts (according to the experiments performed by Schmahmann and Pandya 2006) as basis for the analysis of the FOMs of the vervet monkey brain.

**Fig. 4:**
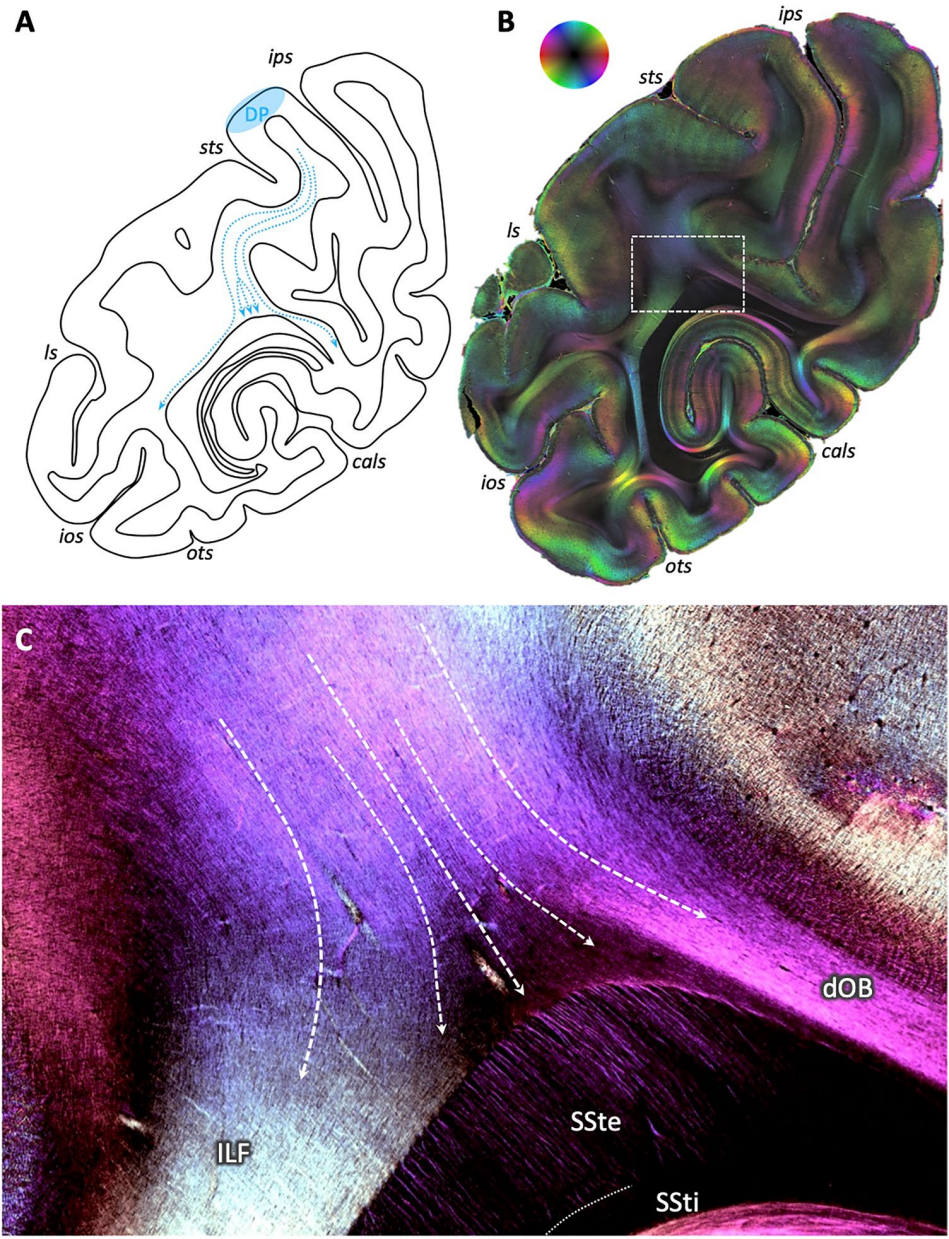
Fibers approaching the dorsal aspect of the sagittal stratum in the vervet monkey brain, in distinction from surrounding fiber bundles. **A** Schematic drawing according to results from Schmahmann and Pandya (2006) illustrating the routes of tracers after injection in area DP (their case 18, p. 245). Note the middle fiber bundle approaching the sagittal stratum. **B** 3D-PLI-derived fiber orientation map at a comparable coronal sectioning plane as in **A**. Region of interest in dorsal sagittal stratum as analyzed in **C** indicated by a white box. Color sphere indicates color coding of fiber orientations. **C** Enlarged, contrast-enhanced depiction of fiber architecture within dorsal sagittal stratum. White arrows indicate the predominant courses of the fibers, with the arrows in the middle following the course of the fibers running towards the dorsal sagittal stratum, intermingling with fibers from out of the sectioning plane (triangle without clearly visible fibers due to their steep inclination). *ls* lunate sulcus. For other conventions, see Fig. 2

Focusing on a section, where fibers from parieto-occipital area DP approached the dorsal part of the SSt in tracer studies (Fig. 4A), accompanied by fibers running in medial and ventral direction, revealed a similar overall fiber direction in 3D-PLI: from dorso-lateral parieto-occipital cortex to the dorsal tip of the SSt (Fig. 4B). Zooming into the transition zone from the deep white matter to the SSt showed that this fiber bundle could be subdivided into three parts (Fig. 4C): a lateral one coursing into the occipito-temporal white matter (color change from blue to light green); a prominent middle part, where fibers kept their direction and intermingled with fibers perpendicular to the sectioning plane (visible by the dark triangular space in between the colored fiber bundles); and a medial bundle joining the fiber tract underneath the IPS, i.e., the dorsal occipital bundle (dOB).

**Fig. 5:**
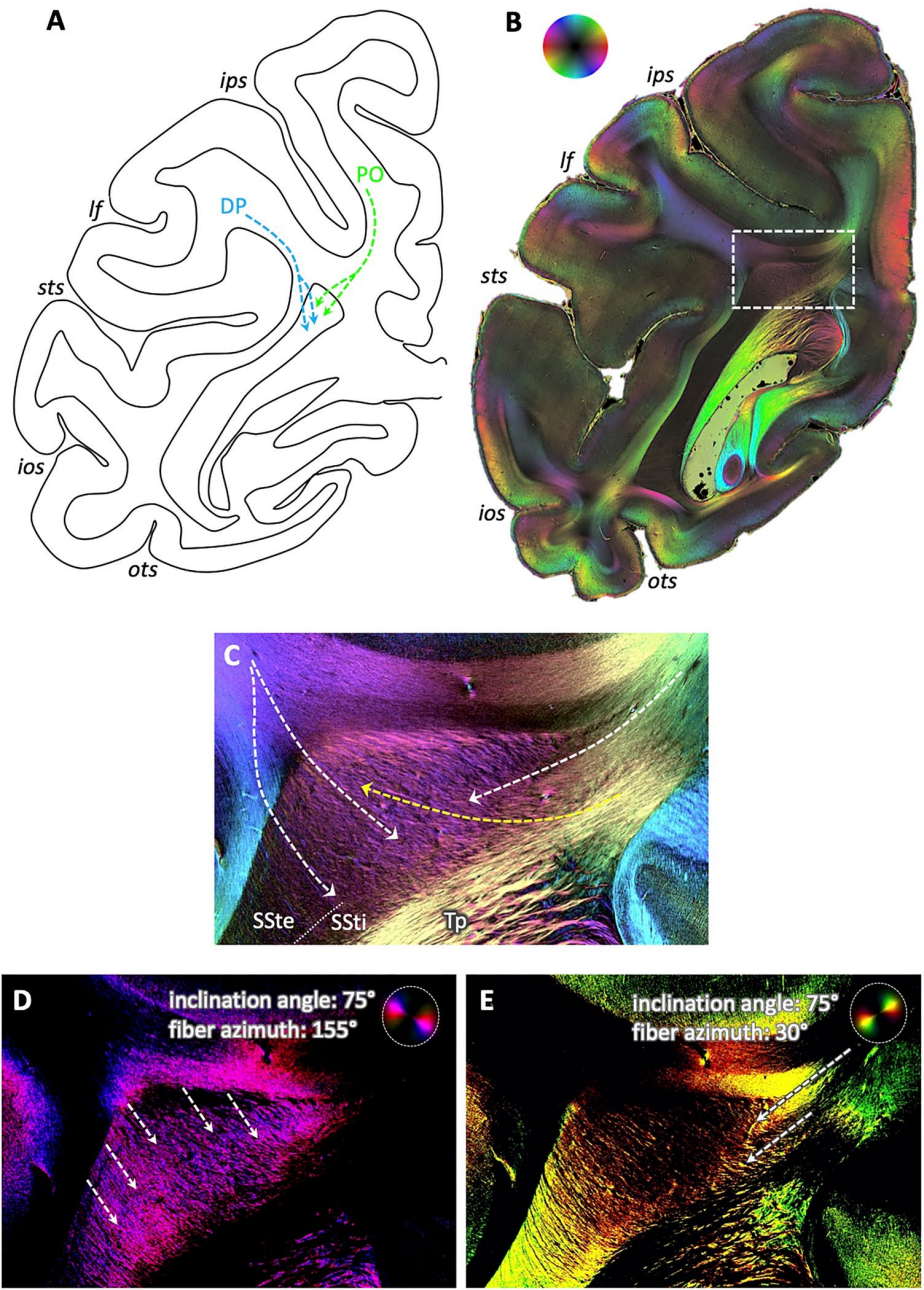
Separate entrance of fibers within the dorsal sagittal stratum of the vervet monkey brain. **A** Schematic depiction of routes of tracers after injection in areas DP (blue arrows) and PO (green arrows), according to Schmahmann and Pandya 2006 (their cases 18 and 17, respectively, p. 245). Note the separate entrance of the fiber bundles into the sagittal stratum. **B** 3D-PLI-derived FOM at a comparable coronal sectioning plane as in **A**. Region of interest in dorsal sagittal stratum as analyzed in **C** indicated by a white box. Color sphere indicates color coding of fiber orientations. **C** Enlarged, contrast-enhanced depiction of fiber architecture within dorsal sagittal stratum. White arrows indicate the predominant courses of the fibers, from top left into the sagittal stratum, reflecting connections from dorsal lateral parieto-occipital cortex (area DP), and from top right, reflecting connections from mesial parietal cortex (area PO). Note the crossing of the transcallosal fibers from the tapetum through the sagittal stratum to the cortex (yellow arrow). **D** and **E** Same enlarged view as in **C**, but as flashlight views of particular fibers of given direction within the section and inclination as indicated by the color coding within the panels. *lf* lateral fissure. For other conventions, see Fig. 2

At a comparable dorsal location, but in a more rostral section, the fibers from DP entered the SSte together with, but clearly separable from the fibers from medial superior parietal area PO according to tracer experiments (Fig. 5A). In 3D-PLI, these separate directions of the fiber bundles from both sides of the IPS were clearly visible (Fig. 5B): while fibers from the IPL were running from latero-dorsal to medio-ventral (blue color), the fiber bundle of the SPL was running perpendicular to it, from medio-dorsal to latero-ventral (green color). Zooming into the dorsal part of the SSt, fibers from different directions intermingled (Fig. 5C): fibers from the SPL entered the SSt at the medial side and changed direction upon entrance (change from green to yellow color and by the flashlight view with only fibers of relevant direction and inclination colored, Fig. 5E). Fibers from the IPL entered from the lateral side, keeping their latero-dorsal to medio-ventral course, as visible by the single fibers colored in blue within the dorsal SSt (particularly in the flashlight view of highlighted fibers in Fig. 5D). These two fiber directions additionally crossed through the tapetum fibers, entering the SSt from the medial side (yellow arrow).

**Fig. 6:**
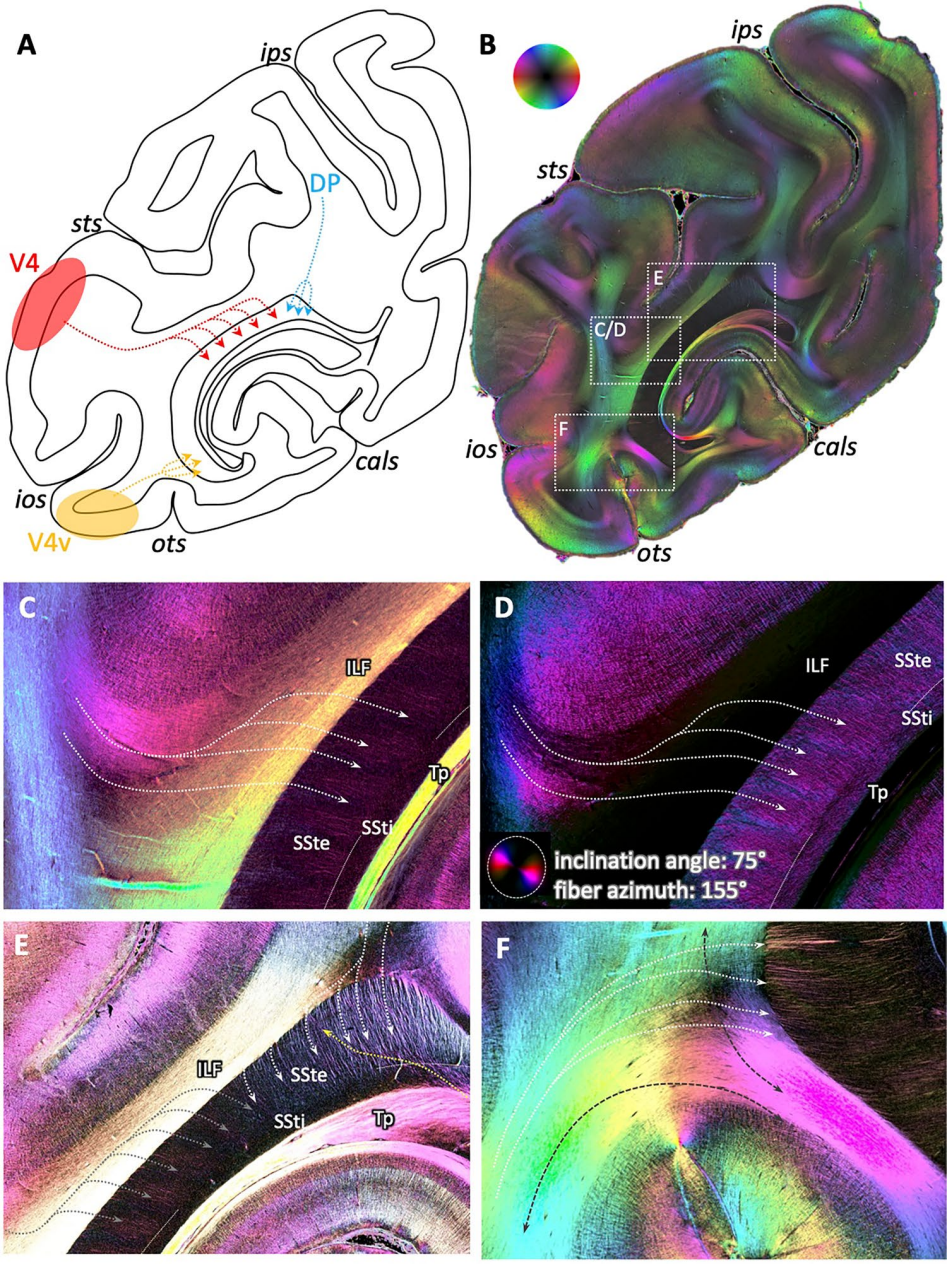
Differential patterns of fiber distribution within the sagittal stratum of the vervet monkey brain depending on origin. **A** Schematic depiction of routes of tracers after injection in areas DP (blue arrows), V4 (red arrows) and V4v (yellow arrows), according to Schmahmann & Pandya 2006 (their cases 18, 20, and 21, respectively, p. 275). Note the patterns of fibers from V4 entering the sagittal stratum in several bundles, in contrast to the locally focused entrance of fibers from DP and V4v. **B** 3D-PLI-derived FOM at a similar coronal sectioning plane as in **A**. White boxes show regions of interest analyzed in **C**–**E**. Color sphere indicates color coding of fiber orientations. **C** Enlarged, contrast-enhanced visualization of the fiber architecture within the middle part of the sagittal stratum. White arrows indicate the predominant direction of fibers coming from lateral occipital cortex, crossing through the ILF (single fibers colored in red), and entering the sagittal stratum in several small parallel bundles (red color in SSte). **D** Same enlarged view as in **C**, but as flashlight view of particular fibers of given direction within the section and inclination as indicated by the color coding within the panels. **E** Enlarged, contrast-enhanced depiction of two groups of fibers with different direction and entering pattern: the small parallel bundles (red color) from lateral occipital cortex (see also **C**) vs. the large bundle from caudal parietal cortex (blue color). **F** Enlarged, contrast-enhanced depiction of fibers from ventral occipital cortex crossing through the ILF and entering the latero-ventral tip of the sagittal stratum

Fibers within the SSt associated with area DP could, furthermore, be distinguished from fibers associated with more ventral areas, e.g., areas V4 and V4v. According to tracer experiments (Schmahmann and Pandya 2006), these fibers enter the SSt either at its ventral tip (area V4v) or with several separate small fiber bundles in its large middle part (area V4; Fig. 6A). Zooming into the respective regions revealed a similar pattern in 3D-PLI (Fig. 6B): fibers from the direction of area V4, i.e., from lateral temporo-occipital cortex bent around the STS, crossed through the ILF (red–orange coloring of the fibers vs. yellow coloring of the ILF) and entered the SSt in small parallel fiber bundles (Fig. 6C, fibers in red color within SSt, particularly visible in the flashlight view, Fig. 6D). These bundles could be clearly separated from those coming from the dorsal aspect of the section and thus from parieto-occipital cortex (Fig. 6E): the latter were not separated into different small bundles, but appeared as one large bundle of many single fibers. Furthermore, the direction differed, coming either from lateral (small fiber bundles colored in red) or from dorsal (fibers colored in light blue). Fibers at the ventral tip of the SSt, contrarily, showed a course oriented from latero-ventral to medio-dorsal (Fig. 6F, fibers colored in red–yellow). Within the underlying white matter, the course of these fibers before they enter the SSte could be determined, parallel to the adjacent U-fibers and crossing through the vertically oriented part of the ILF.

### Exemplary comparison to the human sagittal stratum

**Fig. 7:**
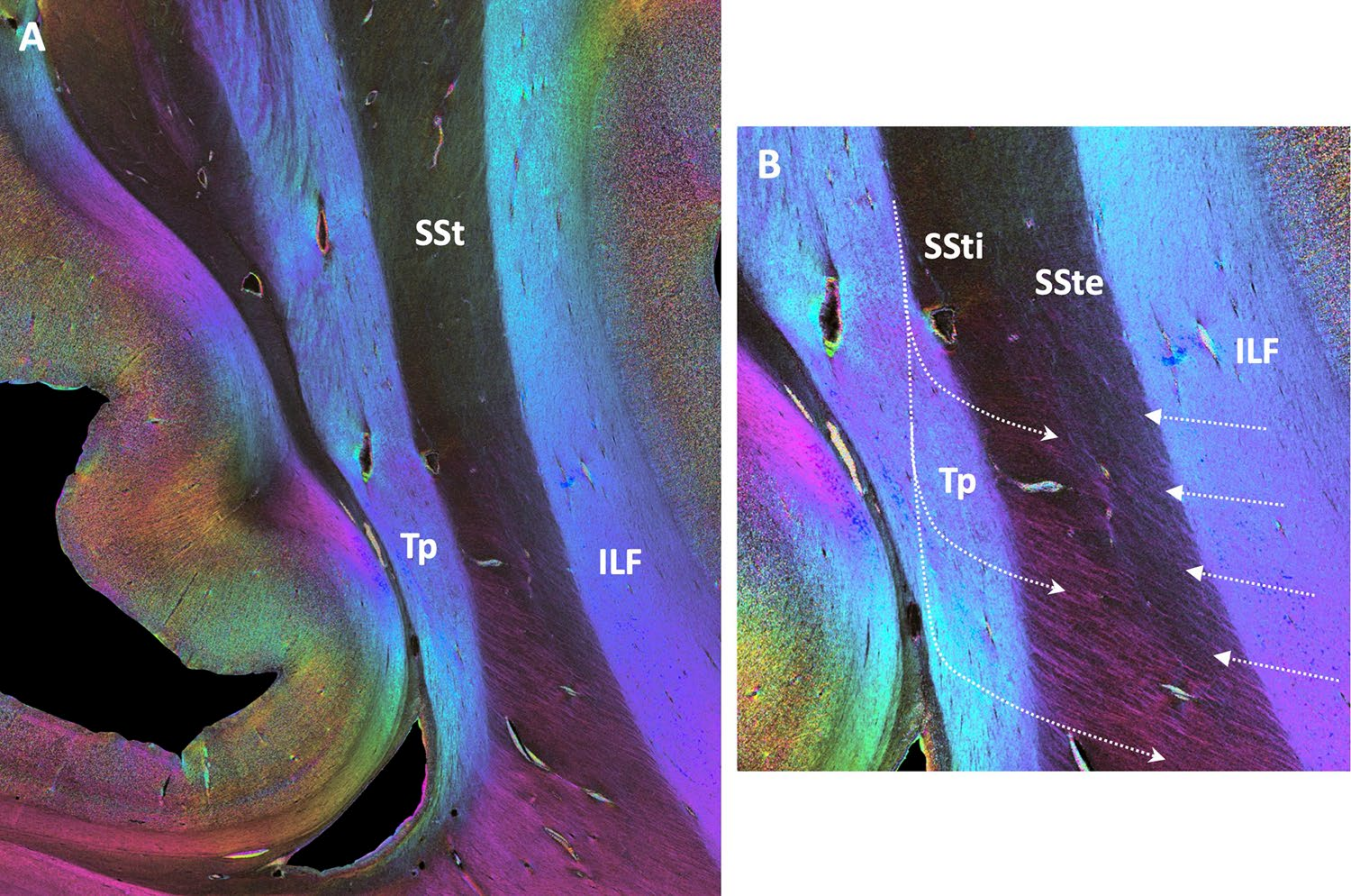
Fiber architecture of the human sagittal stratum. **A** View of the complete sagittal stratum, surrounded by the tapetum (Tp) medially and the deep white matter fiber tracts, e.g., the inferior longitudinal fasciculus (ILF) laterally. **B** Enlarged, contrast-enhanced view of the middle sector of the sagittal stratum, with clear distinction between the external and internal layers (SSte, SSti). Note the fibers coming out of the tapetum and crossing through the complete sagittal stratum in contrast to the fibers entering or leaving the sagittal stratum to the surrounding white matter, which branch and join the SSti (visible by the fading of the colors)

Having demonstrated similar evidence for the overall structure and courses of fibers entering and leaving the SSt in its course through the parieto-occipital white matter in previous tracer experiments and current 3D-PLI analyses in the same non-human primate lineage (macaques and vervets) led back to the question if with the new 3D-PLI technique, a comparable SSt structure might be detectable in humans. The overall appearance of the SSt with a clear delineation from the surrounding white matter and an internal and external layer is indeed comparable (Fig. 7A). Similarly, fibers entering/leaving the layers of the SSt from the tapetum or through the ILF are clearly visible in a high-resolution view (Fig. 7B), suggesting an at least similar or even more complex fiber architecture in humans as compared to non-human primates.

## Discussion

The present study showed that the sagittal stratum in humans and monkeys has a complex fiber structure, beyond the well-known large fiber bundles running in rostro-caudal direction. We could not only clearly show the bilaminar structure of the sagittal stratum, but were able to show, where fibers enter and leave the sagittal stratum to the surrounding white matter on their way from/to different parieto-occipital cortical regions. These patterns as revealed here by 3D-PLI strikingly resembled those found in tracer studies in monkeys.

### Escaping and joining fibers and layering of the sagittal stratum

A bilaminar configuration was found, reflecting two separate rostro-caudal fiber bundles, namely, the optic radiation in the external and the cortico-subcortical projection tracts in the internal layer (Flechsig 1896; Pfeifer 1925; Sachs 1892; Schmahmann and Pandya 2006). Supplementing these studies, the current data showed a clear layering into an external and an internal segment throughout the whole extent of the sagittal stratum. As has been described previously, the layering of the sagittal stratum dissolves in caudal sections close to the occipital pole, as does the sagittal stratum as a whole (Vergani et al. 2014). Approaching the occipital pole region means that less cortical regions were present, until only primary visual area V1 is visible. This phenomenon was particularly pronounced in the vervet monkey brain as V1 covers major parts of mesial and lateral aspects of the occipital lobe. In the human brain, V1 (Brodmann area 17) is mainly confined to the mesial side and only partially encroaches on surrounding occipital lobe at the very caudal end (Amunts et al. 2000). This suggests that only the optic radiation and the external layer of the sagittal stratum are present when approaching the occipital pole. The optic radiation then fans out to reach all parts of V1 and adjacent areas V2 and V3v and V3d (Alvaréz et al. 2015; Wandell and Winawer 2011), resulting in a disappearance of the sagittal stratum, since the fibers are not as parallel as in rostral sections.

The internal layer of the sagittal stratum appears in rostral sections as more and more fibers from occipital, parietal and temporal areas enter the sagittal stratum. These fibers necessarily need to cross through the external layer of the sagittal stratum. This has been clearly shown in tracer experiments in monkeys, where the fibers from a particular brain region can be followed through the white matter as they enter and cross the external layer of the sagittal stratum and join the fiber bundle running within the internal layer on their way to the subcortical target zones (Schmahmann and Pandya 2006). These tracing results have been used as reference in the present study. Shown for different exemplary tracts, the pattern of fiber architecture as revealed by the tracing experiments could be clearly shown by means of 3D-PLI. Of note, not only the global connection could be demonstrated, e.g., that fibers from superior and inferior parietal or ventral occipital cortex enter the sagittal stratum at different dorsal or ventral levels. In addition, the exact distribution of fibers within the sagittal stratum could be reconstructed. This was particularly visible for the fibers originating in the region of area V4 and V4D as compared to those coming from parietal areas. According to tracer experiments (Schmahmann and Pandya 2006), the fibers from V4/V4D enter the sagittal stratum in its middle sector in several parallel interspaced fiber bundles with latero-medial orientation, while the fibers from parietal areas entered the sagittal stratum in its dorsal sector as a large bundle with dorso-ventral orientation. This differential pattern of several interspaced vs. one large fiber bundle could be clearly shown in the 3D-PLI sections of the present study. Thus, without using a tracing technique with injections, 3D-PLI is able to show the exact same connection pattern in the same non-human primate lineage. Providing the same results as the gold standard for studying structural connectivity, e.g., of interspaced separated fiber bundles in the middle of the SSt, which was also reported in the tracer experiments of Schmahmann and Pandya (2006), could be regarded as an external validation of the novel 3D-PLI technique. On the one hand, this facilitates systematic assessments of the complex white matter architecture and the different fiber bundles in one and the same brain (as compared to separate tracer injections in different hemispheres). On the other hand, it enables translating this approach to the human brain, where tracing using injections is ethically not feasible. 3D-PLI results obtained in the human brain could then be more easily understood as adequately revealing the fiber microarchitecture, which is a prerequisite for consecutive comparison and cross-validation with DWI data.

Thus, 3D-PLI could provide the bridge between tracing and diffusion imaging both within and across species (where comparable white-matter occipital fiber architecture can be detected, see, e.g., Kaneko et al. 2020), as the relevant intermediate step (Caspers and Axer 2019) and a valuable complement to comparisons between DWI and tracing experiments which also revealed comparable results (Schmahmann et al. 2007). At this, ex-vivo diffusion MRI serves as an additional relevant component when bridging between the scales (Roebroeck et al. 2019), as it could reach high resolutions on the mesoscale in the range of tens to hundreds of microns in different species (Aggarwal et al. 2015; Bech et al. 2020; Fritz et al. 2019; Liu et al. 2020; Ly et al. 2020; Wang et al. 2020).

It was also visible in the 3D-PLI data that these entering fibers indeed joined the internal layer of the sagittal stratum: while they could be clearly seen within the external layer, they faded out at the transition to the internal layer. This was indicative of fibers turning out of the sectioning plane into a perpendicular course, which in the case of our coronal brain sections pointed at a rostro-caudal trajectory. Following them continuously to their final subcortical target regions is challenging at the moment as fibers running out of the sectioning plane appear black in the 3D-PLI fiber orientation maps. This would be alleviated by 3D tractography in reconstructed stacks of 3D-PLI sections in the future.

We could additionally show that fibers crossed through both the external and internal segment of the sagittal stratum, originating in the medially adjacent tapetum. The tapetum contains the callosal fibers of the occipital and neighbouring parietal and temporal cortex. To reach their target zones within the cortex (Caspers et al. 2015; Clarke and Miklossy 1990), they have to cross through the sagittal stratum. We could show that they could be clearly distinguished from the fibers entering the sagittal stratum from the surrounding white matter, not only because of their major direction, but also by their complete course from leaving the tapetum, intermingling with and crossing through the different fibers of the sagittal stratum to leaving the sagittal stratum laterally.

Results obtained in this study rely on the analysis of one hemisphere of a vervet monkey brain and an exemplary comparison to a human brain section. Thus, future studies with a larger sample size are desirable to further verify the observations on the fiber architecture obtained here.

### Resolving small fiber bundles in human occipital deep white matter

The packing density of fibers within the deep white matter of the human brain is even higher than in the monkey brain. This challenges current in-vivo diffusion imaging approaches, aiming at studying smaller bundles which cross the large fiber tracts (Dell’Acqua and Catani 2012; Jbabdi and Johansen-Berg 2011). This is particularly true within the occipital lobe, where several large fiber tracts run in parallel, mainly in rostro-caudal direction, such as the optic radiation (as part of the sagittal stratum), the inferior longitudinal fasciculus, the callosal fibers within the tapetum, or the inferior and superior fronto-occipital fasciculi (Catani et al. 2003; Cristina et al. 2014; Forkel et al. 2014; Takemura et al. 2020). Using additional MR contrasts, it was possible to identify another relatively large fiber tract perpendicular to them, running in dorso-ventral direction lateral to the sagittal stratum and crossing through the ILF, i.e., the vertical occipital fasciculus (Bugain et al. 2021; Takemura et al. 2016; Yeatman et al. 2014). These large bundles surrounding the sagittal stratum have also been identified in blunt dissections (De Benedictis et al. 2014).

When it comes to the fine structure beyond these large tracts, e.g., within the sagittal stratum, both dissection and diffusion imaging seemed to have reached their limits in terms of resolution: the bilaminar structure of an internal and external sagittal stratum could only partially be identified (Vergani et al. 2014).

Functional in-vivo mappings during neurosurgery suggest that several complex higher order functions (e.g., hemi-agnosia, spatial neglect, known to involve several occipital and parietal cortical regions) were hampered during direct electrical stimulation of the SSt, indicating involvement of different fiber systems intermingling within the SSt (Berro et al. 2021). The current results shed new light on this issue: the fibers enter, leave or cross through the sagittal stratum in very thin bundles or even as single fibers. This is far below the spatial and angular resolution of routine in-vivo diffusion imaging studies, even of high-angular resolution diffusion protocols, when it comes to identifying single fibers or disentangling crossings of such small bundles. Using advanced fiber orientation reconstruction algorithms, such as constrained spherical deconvolution (Tournier et al. 2007), might enable detection of some additional fiber orientations, but averaged across a considerably large volume of a few cubic millimeters.

The analysis of ultra-high-resolution images resulting from 3D-PLI showed that there are indeed small fiber bundles which cross through the inferior longitudinal or vertical occipital fasciculus on their way to or from the sagittal stratum. They could not only clearly be followed as they crossed through the external sagittal stratum to join the internal sagittal stratum, but also as they approached the sagittal stratum and crossed the large white matter tracts surrounding it.

Thus, the additional fiber directions as visible in the diffusion imaging data (Fig. 1C, D) might indeed hint at such anatomical fine structure of the sagittal stratum. Considering the reconstructed fiber orientations from the diffusion signal alone would prevent any distinction between a correct depiction of the underlying anatomy or false positives due to noise in the data (see, e.g., Leuze et al. 2021). Combination with an ultra-high-resolution technique such as 3D-PLI may provide detailed insights into the fiber architecture of the brain by decoding the local fiber configurations (Caspers and Axer 2019). This particularly holds true for regions with one major fiber direction dominating the local architecture, such as the sagittal stratum, which could lead to neglect of the minor directions. Based on the examples shown here for the human brain with a highly similar pattern of fiber architecture as found in the vervet monkey, future studies are needed to follow the individual fiber tracts which join the internal layer of the sagittal stratum to reach their subcortical target regions. This might be facilitated in the future by fiber tractography based on reconstructed stacks of 3D-PLI sections, based on aggregated 3D-PLI-derived fiber orientation distribution functions (Alimi et al. 2020; Axer et al. 2016; Reckfort et al. 2015; Schmitz et al. 2018), in combination with ultra-high-field ex-vivo DWI (Aggarwal et al. 2015; Fritz et al. 2019; Ly et al. 2020), as has been shown, e.g., for the trigeminal pathway in the human brainstem (Henssen et al. 2019). With ex-vivo DWI being inherently 3D when applied to a whole tissue volume, it could serve as relevant reference for the tractography data derived from 3D-PLI. With the high resolution of 3D-PLI of about one micrometer in-plane across species, these approaches are mutually complementary and could thus be used in combination in the future to understand the complex multi-level fiber architecture of the brain.

Similar to the knowledge gained from respective tracing experiments in monkeys, this high-resolution fiber tract analysis in humans is of particular importance to understand which parts of occipital and adjacent parietal and temporal cortices are connected with which subcortical structure, e.g., within the thalamus or among the pontine or mesencephalic nuclei. This will enable a better understanding of cortico-subcortical feedback loops for top-down control of incoming information from the subcortical relay stations as well as information flow to subcortical control centers.

## Conclusion

Using 3D-PLI as a method for ultra-high-resolution mapping of fibers, we could demonstrate the complex architecture of in- and outgoing as well as crossing fibers of the sagittal stratum. Showing this in monkeys, for which data from tracing experiments are available, enabled a direct comparison with the gold standard for studying structural connectivity in the brain, and thus, external validation of the present results. The translation to the human brain using the same technique finally revealed a comparably complex fiber architecture of the sagittal stratum. These insights could be used for understanding the structure of the fiber orientation distributions of the in-vivo high-angular resolution diffusion imaging data as revealed by spherical deconvolution: our study hints at interpreting the small handles of the glyphs potentially as more than noise and might indeed reveal additional fiber directions beyond the very dominant rostro-caudal ones. Taking this as a starting point could help for future studies, in which the advantages of both diffusion imaging and 3D-PLI might be used for mutual information to understand the complex local situation of crossing and intermingling fibers.

## Data Availability

Data and materials can be made available upon reasonable request.

## Code availability

The manuscript does not contain any specific code to be shared.
